# Optimizing Skin Cancer Survival Prediction with Ensemble Techniques

**DOI:** 10.3390/bioengineering11010043

**Published:** 2023-12-31

**Authors:** Erum Yousef Abbasi, Zhongliang Deng, Arif Hussain Magsi, Qasim Ali, Kamlesh Kumar, Asma Zubedi

**Affiliations:** 1 State Key Laboratory of Wireless Network Positioning and Communication Engineering Integration Research, School of Electronics Engineering, Beijing University of Posts and Telecommunications, Beijing 100876, China; dengzhl@bupt.edu.cn; 2 State Key Laboratory of Networking and Switching Technology, Beijing University of Posts and Telecommunications, Beijing 100876, China; ahmagsi@bupt.edu.cn; 3 Department of Software Engineering, Mehran University of Engineering and Technology, Jamshoro 76062, Pakistan; qasim.arain@faculty.muet.edu.pk; 4 School of Electronics Engineering, Beijing University of Posts and Telecommunications, Beijing 100876, China; kamleshsoothar@gmail.com; 5 School of Economics and Management, Beijing University of Posts and Telecommunications, Beijing 100876, China; asmazubedi@bupt.edu.cn

**Keywords:** skin cancer, melanoma, machine learning, ensemble technique, feature selection

## Abstract

The advancement in cancer research using high throughput technology and artificial intelligence (AI) is gaining momentum to improve disease diagnosis and targeted therapy. However, the complex and imbalanced data with high dimensionality pose significant challenges for computational approaches and multi-omics data analysis. This study focuses on predicting skin cancer and analyzing overall survival probability. We employ the Kaplan–Meier estimator and Cox proportional hazards regression model, utilizing high-throughput machine learning (ML)-based ensemble methods. Our proposed ML-based ensemble techniques are applied to a publicly available dataset from the ICGC Data Portal, specifically targeting skin cutaneous melanoma cancers (SKCM). We used eight baseline classifiers, namely, random forest (RF), decision tree (DT), gradient boosting (GB), AdaBoost, Gaussian naïve Bayes (GNB), extra tree (ET), logistic regression (LR), and light gradient boosting machine (Light GBM or LGBM). The study evaluated the performance of the proposed ensemble methods and survival analysis on SKCM. The proposed methods demonstrated promising results, outperforming other algorithms and models in terms of accuracy compared to traditional methods. Specifically, the RF classifier exhibited outstanding precision results. Additionally, four different ensemble methods (stacking, bagging, boosting, and voting) were created and trained to achieve optimal results. The performance was evaluated and interpreted using accuracy, precision, recall, F1 score, confusion matrix, and ROC curves, where the voting method achieved a promising accuracy of 99%. On the other hand, the RF classifier achieved an outstanding accuracy of 99%, which exhibits the best performance. We compared our proposed study with the existing state-of-the-art techniques and found significant improvements in several key aspects. Our approach not only demonstrated superior performance in terms of accuracy but also showcased remarkable efficiency. Thus, this research work contributes to diagnosing SKCM with high accuracy.

## 1. Introduction

In recent years, the alarming surge in malignant diseases has become a critical global health concern. Among these malignancies, skin cutaneous melanoma cancer (SKCM) is one of the most aggressive variants, demanding thorough investigation and understanding [1]. According to an International Agency for Research on Cancer (IARC) report, cancer is the leading cause of mortality. The report exhibits that nearly 10 million deaths have resulted from various types of cancer [2]. The World Health Organization (WHO) 2023 report illustrates that cancer is the second leading cause of death (16%), followed by cardiovascular disease (27%) [3]. In such a situation, the early diagnosis of a disease can cure and prevent the patients from further jeopardy. In general, there are two main forms of skin cancer: melanoma (cancers resulting from melanocyte malfunction) and non-melanoma skin cancers (from cells generated from the epidermis) [4]. Among various types of cancers, SKCM has become one of the most prevalent cancers in the last ten years [5] with tumors made of melanocyte cells. It is currently a major public health issue worldwide, and the increasing prevalence of the disease might significantly impact the world’s population and economy [6]. However, early diagnosis and effective tumor therapy lead to a cure rate of over 90% in individuals with incipient melanoma [7]. There are several factors for an increased number of skin cancers. One of the most common occurrences of skin cancer is due to ultraviolet (UV) rays [8]. Other reasons include sun exposure, depletion of the ozone layer, genetic predisposition, and so on.

Several studies have shown that SKCM results from abnormalities in transcriptional and epigenetic factors, including the expression of messenger ribonucleic acid (mRNAs) and micro ribonucleic acid (miRNAs), the aberration in methylation patterns of CpG islands of genes, and histone modifications, which opens the door for the development of potential molecular biomarkers in melanoma [9,10]. As predictive indicators for cutaneous melanoma, miRNA expression has been implicated in several past studies.

**Figure 1:**
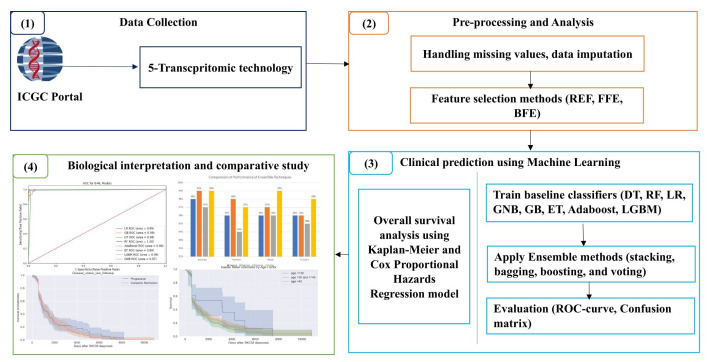
Workflow of the proposed research.

Various healthcare sectors, including dermatology, have leveraged artificial intelligence (AI), revolutionizing diagnostic and therapeutic processes. Diverse biomedical data from health records, medical images, IoT sensor data, and text can be used to predict SKCM. Specifically, machine learning (ML) and deep learning (DL) significantly contribute to predicting the disease on publicly available datasets. The most recent skin cancer detection technique includes dermoscopy with AI, which leverages the handheld device for magnifying the skin and allows dermatologists to examine moles and lesions in detail. The ML and DL algorithms require structured data for classification, with lower prediction accuracy, and require more computational time. Due to its superiority over traditional analytical methods, AI has significantly uplifted the healthcare industry. Applications of AI in healthcare are being used with increasing optimism, and they range from speeding up the research of new drugs to helping with medical diagnosis, treatment, and administrative support. Additionally, using it as an adjuvant in clinical decision making can be advantageous [11,12]. Various ML algorithms are leveraged to predict different diseases in the early stage after diagnosing different attributes of the disease. Those diseases include different cancer types, diabetes, kidney disease, and other diseases [13]. Specifically, ensemble learning is a learning method that combines multiple baseline models to create a powerful single model. It reduces overfitting risk and has been successfully applied in various fields. Common ensemble techniques include averaging, bagging, boosting, stacking, and voting [14]. Traditional ensemble learning integrates ML models across various fields, but efforts have shifted to DL, focusing on complex models and integrating them across various fields [15]. The other recent skin cancer detection technique involves reflectance confocal microscopy (RCM), a non-invasive imaging approach, that enables high-resolution skin imaging at the cellular level. It aids dermatologists in visualizing skin structures and identifying abnormal cells without a biopsy procedure. Another recent technique leverages smartphone applications where mobile applications utilize smartphone cameras for skin self-examination. These apps often use AI algorithms to analyze photos and provide risk assessments. The main objective of this study is to propose four ensemble methods for predicting skin cancer by utilizing ML algorithms. We experimented with five transcriptomic technologies from the ICGC portal [16]. In this research, three features were leveraged—recursive feature elimination (RFE), forward feature selection (FFE), and backward feature elimination (BFE)—for the ensemble method. This paper discusses the five phases for ensemble methods based on ML algorithms that use transcriptomic technology data to predict SKCM. Figure 1 illustrates the workflow of our integrative study. Figure 1 (1) depicts the data collection source and 5 different transcriptomic technology datasets. Figure 1 (2) illustrates preprocessing and analysis steps. We handled missing data with the MICE imputation technique and applied three methods for best feature selections. Figure 1 (3) represents the experimental achievements of our study using different ML algorithms as baseline classifiers to create an ensemble method. Overall survival was analyzed with the Kaplan–Meier and Cox hazard regression model. Different from traditional methods in existing literature, this study contributes to predict skin cancer using various ML techniques. The novelty of this work lies in its comprehensive approach, combining high-throughput ML-based ensemble methods with the analysis of multi-omics data, particularly addressing the challenges posed by complex and imbalanced datasets with high dimensionality. Figure 1 (4) shows the biological interpretation and comparative study.

The key contributions of this study are as follows:

- We evaluated various techniques for SKCM prediction considering their suitability and effectiveness in this context.
- We used RFE, FFE, and BFE features for ensemble methods.
- We analyzed the overall survival (OS) analysis and progression through the Kaplan–Meier estimator and the Cox hazard proportional regression model.
- We used eight baseline classifiers, namely, random forest (RF), decision tree (DT), gradient boosting (GB), AdaBoost, Gaussian naïve Bayes (GNB), extra tree (ET), logistic regression (LR), and light GBM in this research work.
- We applied ML algorithms for predicting the disease with various selected features.
- We trained four ensemble learning methods, including stacking, bagging, boosting, and voting, to achieve the best results.

The rest of the article is organized as follows: Section 2 provides a detailed description of related work. In Section 3, we provide ML-based materials and methods that include data collection, preprocessing techniques, and classification methods. Section 4 exhibits the achieved results and discussion. Finally, Section 5 concludes the article.

## 2. Related Work

In recent years, cancer has been a very undeniable global health challenge. There are various cancer types, such as lymphoma, leukemia, breast cancer [17], lung cancer, skin cancer, and so on. Early skin cancer detection significantly impacts prognosis, and various techniques have been exploited. From histopathological examination to advanced imaging modalities [18], the quest for optimizing predictive models has given rise to the integration of ensemble techniques. This literature review delves into the multifaceted landscape of skin cancer detection methodologies, focusing on the evolving role of ensemble techniques in enhancing survival prediction accuracy. On the other hand, ML is greatly contributing to anomaly detection in various fields, including health care, vehicular networks [19,20], the Internet of Things (IoT), E-commerce, and so on. ML and DL algorithms significantly aid in identifying skin cancer, with early detection potentially leading to successful treatment, making melanoma a significant health concern [21,22,23,24]. Various ML and DL techniques have been applied in existing literature, such as in [25], where the authors presented a convolutional neural network (CNN) based DL stacked ensemble framework for melanoma skin cancer detection using transfer learning. The model uses multiple CNN sub-models and a meta-learner to predict malignant melanoma moles. The model achieves a high accuracy of 95.76%, precision of 95.60%, recall of 96.67%, specificity of 94.67%, F1 score of 94.67%, and area under the curve (AUC) of 0.957% identifying both benign and malignant melanoma. Although this research is important, it could not achieve better accuracy. Similarly, another work in [26] proposed a DL-based skin cancer detection system on an imbalanced dataset. The authors employed the MNIST: HAM10000 dataset that contains seven classes of skin lesions. In order to classify the skin cancer, the authors utilized AlexNet, InceptionV3, and RegNetY-320 techniques. However, the achieved accuracy (91%), F1-score (88.1%), and ROC curve (95%) reflect a poor accuracy as compared to our proposed study.

Moreover, the authors in [27] proposed a CNN-based skin cancer detection system using a publicly available dataset, HAM10000, that includes seven skin cancer types. The authors achieved the following: accuracy (86%), precision (84%), recall (86%), and F-1 score (86%). Thus, all the achieved results fall in the 80s, which reflects the poor performance of the proposed study. Authors in [28] employed a CNN-based approach using a HAM10000 dataset that comprises 6705 benign and 1113 malignant samples and 2197 unknown lesion samples. The proposed model achieved an accuracy of 93.16% on training and 91.93% on testing. Moreover, the authors balanced the dataset of both classes, resulting in an enhanced accuracy of categorization. Despite training several transfer learning models on the same dataset, the outcomes did not surpass those of their proposed model. Another similar work in [29] proposed a CNN-based skin cancer diagnosis that is evaluated using the ISIC 2019 dataset. This work is based on multiclassification system that classifies the cancer types including benign keratosis, melanoma, melanocytic nevi, and basal cell carcinoma. The achieved results depicted an accuracy of 96.91%, which is inefficient as compared to our proposed study. Similar to our work, authors in [9] studied three immune-related mRNAs (SUCO, BTN3A1, and TBC1D2) linked to melanoma prognosis. This study used univariate Cox regression and Kaplan–Meier analysis to compare the overall survival probability between high-risk and low-risk groups, analyzing the time-dependent ROC curve. However, the accuracy of various classifiers is lower as compared to our achieved results.

Furthermore, reference [10] developed a combination of ML and DL-based tools to predict the short-term survival of cutaneous malignant melanoma (CMM), a common malignancy. The study found that additional clinical variables such as sex, tumor site, histotype, growth phase, and age were significantly linked to overall survival, with DNN and RF models showing the best prognostic performance with an accuracy of 91% and 88%, respectively. Reference [30] analyzed mRNA expressions of m5C regulators in colorectal cancer tissues and identified high mutation frequency. NOP2 and YBX1 were highly expressed in prostate, gallbladder, lung, and renal cancers. NSUN6 functions as a tumor suppressor in pancreatic cancer. UV radiation was identified as the primary environmental driver. The authors in [31] trained a HAM10000 ISIC dataset using DL for multiclass skin cancer diagnosis. The proposed model detects the skin lesion with an accuracy of 96.26%.

### Limitations of Existing Studies

Skin cancer has become an interesting topic in current research. Most past studies preferred survival analysis using KM and Cox proportional hazards regression model. Unlike those traditional models, our study proposes ensemble methods for predicting SKCM and analyzing the survival probability using the KM and Coz hazard regression model. Table 1 demonstrates the limitations of previous studies in comparison with our proposed research.

Table 2 depicts the notations and their descriptions used in this paper.

## 3. Materials and Methods

This study focuses on overall survival analysis and ensemble methods to predict SKCM and is described as an integrative omics study in this paper. The suggested integrative model generates trained ML classifiers that can be utilized as SKCM prediction and feature selection strategies. Our proposed research methodology involves various steps, as mentioned below:

### 3.1. Dataset Collection

**Figure 2:**
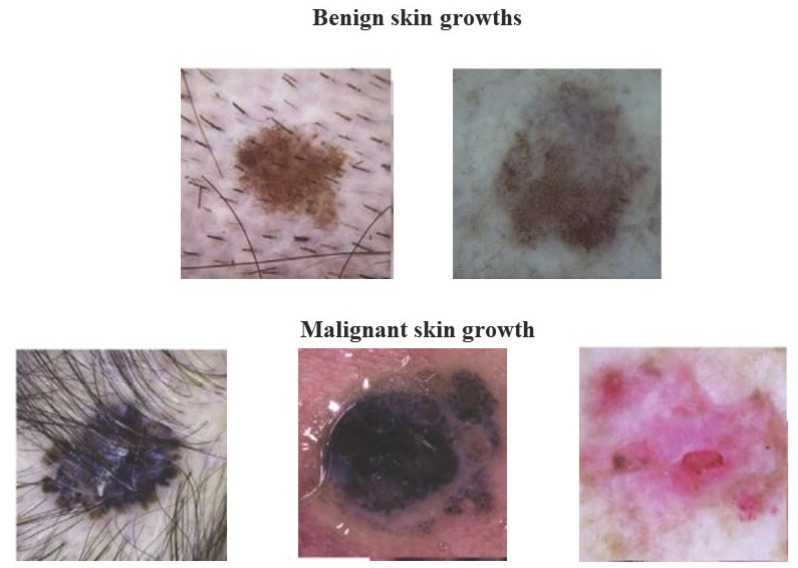
Various skin samples.

We initially collected a dataset from a publicly available source [16] to propose ensemble methods. As illustrated in Table 3, There were five categories of multi-omics data in the datasets: donor, simple somatic mutation, miRNA seq, copy number somatic mutation, and specimen. Our cohort of 471 patients includes information on the patient’s history, such as age, gender, length of survival, and donor relapse type. There are 377,735 samples in the copy number somatic mutation file and 369,409 samples in the miRNA seq file, all of which were examined and approved by the Illumina HiSeq verification platform. There are 1,048,575 samples in the simple somatic mutation file, which were examined and verified by Illumina GA sequencing and the Illumina HiSeq platform. There are 947 samples in the specimen file. Figure 2 shows the benign and malignant samples.

Table 3 illustrates the detailed description of the dataset.

**Figure 3:**
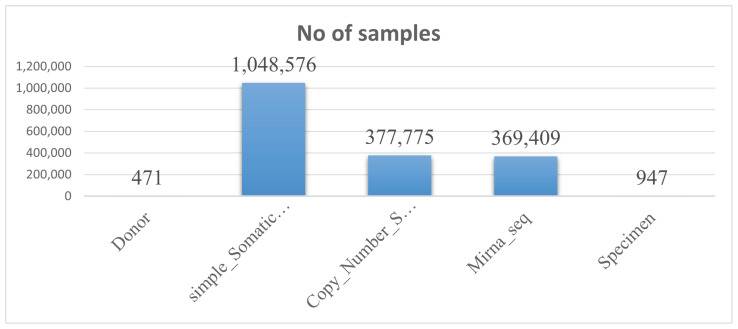
Dataset description.

Figure 3 depicts the detailed dataset description in graphical form as below.

### 3.2. Preprocessing

The data preprocessing plays a significant role in achieving better accuracy results in ML. Considering the importance of preprocessing, we applied various preprocessing techniques, including removing noisy data, dividing the dataset into training and testing, and feature selection. The detail of each technique is elaborated below. Initially, we removed unreliable noisy data. The features with missing value scores of more than 70% and less than 10% were excluded. The features with more than 10% and less than 70% of the data missing score were included [32]. Missing data were imputed using the multiple imputation chained equation (MICE) technique that applies the k-neighbor algorithms criteria [33]. A widely recognized Python programming language at an advanced level was employed in this research paper. These preprocessing techniques collectively contribute to the enhancement of model accuracy and robustness. Removing noisy data and selecting relevant features ensure that the subsequent machine learning models are trained on a cleaner and more informative dataset, ultimately leading to improved predictive performance. Furthermore, the impact of these preprocessing techniques on the results is noteworthy. By systematically cleaning the data and selecting features judiciously, we mitigate the risk of model overfitting and improve generalization to new, unseen data.

#### Feature Selection

Precision Health uses statistical modeling based on clinical and biological data to predict patient outcomes more accurately. Traditional approaches struggle with large datasets, leading to feature selection research in various fields [34]. The following approaches improve model performance, deliver features quickly and cost-effectively, facilitate data visualization, and offer a better understanding of the data-generating process. For solving and reducing the difficulty of learning tasks, feature selection aims at removing irrelevant or redundant features. For selecting the best features, we have applied three different feature selection methods, which are discussed below:

**a. Forward Feature Elimination Method (FFE)**: The FFE method is the reverse of the backward elimination method, starting with empty features and adding them one by one until any excluded features can significantly contribute to the model’s outcome. The most significant feature is added first, and the model is refitted with the new feature. Test statistics or *p* values are recomputed for all remaining features. The features with the largest test statistic are chosen from the remaining features and added to the model [35]. Suppose *X* is an input set of features with n size of features that can be defined using Equation (1). Initially, we have an empty set of features $X_{0}=\emptyset \;$ with the t size of the subset, and it is initialized with a null set, and t=0 where t denotes the size of subset features, and it can be defined using Equation (2). After initializing the input variable, we have a subset of features $Z_{t}=z_{b|b=1,2,3,\ldots ,t;\;Z_{b}\in X}$ based on which the method refits the features. Let us define the subset of features using Equation (3). In Equation (4), assume $p^{+}$ to be the features that will find the arg max $\left(Z_{t}+z\right)$ here $z\in X-Z_{t}$ and maximize our selection criteria, which are associated with the classifier having the best score; score can be accuracy, mean absolute error (MAE), residual square $R^{2}$ on the output set of features that is $Z_{t}$. This process continues until we get the desired set *h* of features with a good score. The iterative process is described by Equations (5)–(7). Equation (5) updates the feature subset $Z_{t+1}$ by adding the most significant feature $p^{+}$ to the existing subset $Z_{t}$. Equation (6) increments the variable *t* to continue the iterative process, and Equation (7) marks the termination of the process when *t* reaches the desired set of features *h*.

(1) $$\begin{array}{c} \begin{array}{c} X=\{X_{1},X_{2},X_{3},\ldots ,X_{n}\} \end{array} \end{array}$$

(2) $$\begin{array}{c} \begin{array}{c} X_{0}=\emptyset ,\;t=0 \end{array} \end{array}$$

(3) $$\begin{array}{c} \begin{array}{c} Z_{t}=\left\{z_{b}|b=1,2,3,\ldots ,t;\;z_{b}\in X\right\},\;\mathrm{where}\;t=\left\{1,2,3,\ldots ,n\right\} \end{array} \end{array}$$

(4) $$\begin{array}{c} \begin{array}{c} p^{+}=\arg\max\left(Z_{t}+Z\right)\mathrm{where},z\in X-Z_{t} \end{array} \end{array}$$

(5) $$\begin{array}{c} \begin{array}{c} Z_{t+1}=Z_{t}+p^{+} \end{array} \end{array}$$

(6) $$\begin{array}{c} \begin{array}{c} t=t+1 \end{array} \end{array}$$

(7) $$\begin{array}{c} \begin{array}{c} t=h \end{array} \end{array}$$

Algorithm 1 shows the process of the forward selection method. We have input features *X*, and we want the best features. These features will be selected based on the value of score $Z_{t}$. This method starts with an empty set and then fits the model with a good score; simultaneously, features will be added and updated. This process will terminate when we get the desired set of features.
**Algorithm 1** Forward feature elimination.1: **Input:** $X=\{X_{1},X_{2},X_{3},\ldots ,X_{n}\}$2: **Output:** $Z_{t}=\left\{z_{b}|b=1,2,3,\ldots ,t;Z_{b}\in X\right\}$3:**Start**4:     Prepare an empty array using (1)5:     Evaluate the fitness of the best feature using (4)6:     **IF** $Z_{t}+p^{+}>X_{0}$7:          Update the features using (5)8:     **End If**9:     t=t+110:   Repeat Step 2.11:   Terminate using (7)12:**End**

**b. Backward Feature Elimination Method (BFE)**: BFE is a simple feature selection method that starts with a full model and deletes features until all remaining features have significant contributions. The least significant feature is deleted first, followed by refitting the model without the deleted feature and recompiling test statistics [36]. To understand the workings of this method, we have a set of features *A* with r size of dimensions that can be interpreted using Equation (8). We initialize the method using Equation (9) with a given set of features. Once the input variable is initialized, we have a subset of features $D_{w}$ based on which method refits the features using Equation (10). Assume ${T\;}^{-}$ to be the features that will find the arg max $\left(D_{w}-g\right)$ where $g\in A-D_{w}$ maximize our selection criteria that is associated with the classifier having the best score; score can be accuracy, MAE, r2 on the set of features that is $D_{w}$. This process continues until we have the desired set *L* of features with a good score. Equations (11)–(14) detail the steps involved in the iterative feature elimination process.

(8) $$\begin{array}{c} \begin{array}{c} A=\{A_{1},A_{2},A_{3},\ldots ,A_{r}\} \end{array} \end{array}$$

(9) $$\begin{array}{c} \begin{array}{c} \;B_{0}=A,\;w=r \end{array} \end{array}$$

(10) $$\begin{array}{c} \begin{array}{c} D_{w}=\left\{g_{i}|i=1,2,3,\ldots ,w;\;g_{i}\in A\right\},\;\mathrm{where}\;w=\left\{1,2,3,\ldots ,m\right\} \end{array} \end{array}$$

(11) $$\begin{array}{c} \begin{array}{c} T^{-}=\arg\max\left(D_{w}-g\right),\;\mathrm{where}\;g\in A-D_{w} \end{array} \end{array}$$

(12) $$\begin{array}{c} \begin{array}{c} D_{w+1}=D_{w}-T^{-} \end{array} \end{array}$$

(13) $$\begin{array}{c} \begin{array}{c} w=w+1 \end{array} \end{array}$$

(14) $$\begin{array}{c} \begin{array}{c} W=L \end{array} \end{array}$$

Algorithm 2 shows the process of the backward selection method. This method takes the full set of input features, calculates the score of classifiers, takes the features with good results, iteratively repeats step 3 until it achieves the desired number of features, and then terminates.
**Algorithm 2** Backward feature elimination.1:**Input:** $A=\{A_{\left(1\right)},A_{\left(2\right)},A_{\left(3\right)},\ldots ,A_{r}\}$2:**Output:**$D_{w}=\left\{g_{i}|i=1,2,3,\ldots ,w;\;g_{i}\in A\right\}$3:**Start**4:     Begin with the full set of input features using (8)5:     Evaluate the fitness of the best feature using (10)6:     **IF** $D_{w}-T^{-}>B_{0}$7:          Update the features using (11)8:          w=w−19:     **End If**10:   Repeat step 211:   Terminate using (14)12:**End**

**c. Recursive Feature Elimination (RFE)**: RFE is a method that selects the optimal feature subset based on the learned model and classification accuracy. We have calculated the feature importance using the training RF model. Algorithm 3 describes the process of RFE. This method works as a ranking procedure.
**Algorithm 3** Recursive feature elimination.1:**Input:**2:     a. Training set *W*3:     b. Set of *C* features $M=\{M_{\left(1\right)},M_{\left(2\right)},\ldots ,M_{C}\}$4:     c. Ranking Method N(W,M)5:**Output:**6:     Ranking *J*7:**Start**8:     Initialize training set *W*9:     **Repeat for** *i* in {1:C}10:          Set the Rank *C* using N(W,M)11:          M∗← last ranked feature in *M*12:          J(C−i+1)←M∗13:          M←M−M∗14:**End**

### 3.3. Proposed Methodology

Unlike traditional research for detecting and predicting SKCM disease, our proposed research exploits ensemble methods (stacking, bagging, boosting, and voting). The proposed research includes the latest ensemble methods to predict SKCM disease using various ML classifiers and analyze the overall survival using the Kaplan–Meier and Cox proportional hazards regression models. To train ensemble methods, we initially create and train baseline classifiers (RF, GB, NB, LR, ET, AdaBoost, DT, LGBM). The performance was evaluated for accuracy, precision, recall, and F1 score. ROC curve and confusion matrix were generated to illustrate the performance. Following is the detail of baseline classifiers.

**RF:** The RF is a highly powerful ML classifier, which amalgamates diverse DT outputs using a majority voting mechanism. This technique increases the resilience of the solution, specifically in challenging problem domains. The overall prediction is derived by computing the average of the results generated by individual DTs.

**GB:** It is a powerful ensemble learning ML classifier. Unlike RF, which combines various DTs, the GB creates a sequential DT, with each subsequent tree correcting the errors. The classifiers optimize a loss function by iteratively adding weak learners, typically shallow DT, to the ensemble. Each tree is trained to emphasize the instances where the model performs poorly, gradually refining the overall predictive capability. The GB is known for its high predictive accuracy and adaptability to various data types, making it a popular choice for classification and regression tasks.

**NB:** It is a simple classifier that leverages Bayes’ theorem for predicting the unlabeled data points. It involves the computation of previous probabilities related to various classes and their application to the latest data. The simplicity and computational efficiency of GNB arise from the assumption of feature independence, making it a streamlined approach for classification tasks.

**LR:** It is used to predict the probability of the categorical data. LR utilizes a logistic function for calculating the probability in binary classification, where the output is dichotomous, representing two classes. It is also named the sigmoid function, which transforms the linear combination of input features into a value between 0 and 1, signifying the likelihood of belonging to a particular class. This makes LR particularly well-suited for problems with binary outcomes, such as in spam detection or medical diagnosis.

**ET:** It is an ensemble learning method related to the DT algorithm. Similar to RF, ET develops a forest of DTs for prediction. In ET, for each split of the DT, the ETs randomly select the feature to split on, leading to a higher level of diversity among individual trees in the ensemble. This increased randomness often results in a more robust model and can be particularly useful in mitigating overfitting. ET is famous for its efficiency and accuracy in handling high-dimensional data, making it a valuable classifier.

**AdaBoost:** This is a popular ensemble learning classifier for regression and classifications. It combines multiple weak learners’ predictions to create an efficient and accurate prediction model. The algorithm assigns weights to each data point, and, in each iteration, it focuses on the misclassified instances, adjusting their weights to prioritize correct classification in the subsequent iteration.

**DT:** It is a highly recognized ML algorithm that evaluates the samples to categories as per their feature values. The DT creation process entails evaluating training samples and considering the most reliable features to partition the data into subsets, guided by principles like information gain or the Gini index. The motive is to create a tree capable of precisely predicting outcomes for new data based on the available features.

**LGBM:** This is an effective gradient-boosting framework exploited for advanced ML tasks. Unlike traditional GB methods, it uses a “leaf-wise” tree growth approach. This technique follows to expand the structure of the tree, integrating leaves that result in the maximum reduction of the loss function, ultimately leading to faster training times.

Algorithm 4 interprets the baseline classifiers and ensemble methods training in general. It depicts the working of ensemble models. Initially, we train base algorithms such as RF, DT, NB, GB, LGBM, LR, and AdaBoost. Then we train four ensemble methods that include stacking, bagging, boosting, and voting. Here, meta-algorithm H denotes the ensemble methods.

**Figure 4:**
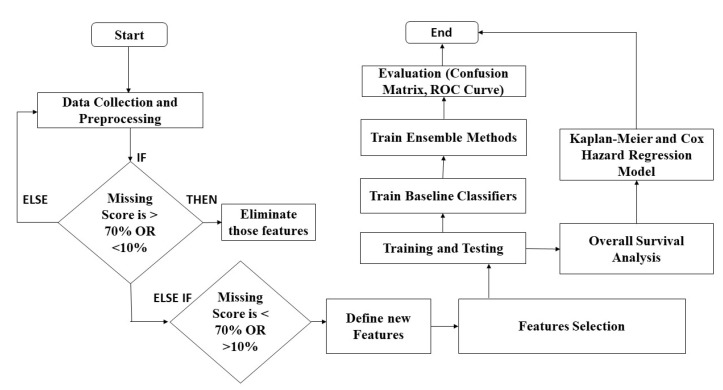
Flow chart of proposed study.

Figure 4 demonstrates the flow of our study. After data collection, the features with missing scores greater than 70% and less than 10% will be eliminated. Any features with missing scores less than 70% and greater than 10% will be included, and a new set of features will be defined. For selecting the best features among newly defined features, we applied three different feature selection methods: REF, FFE, and BFE. Our study evaluates four ensemble methods and an overall survival analysis using the Kaplan–Meier estimator and the Cox hazard regression model. To create ensemble methods, we must first create and train baseline classifiers.
**Algorithm 4** ML-based ensemble methods.1:**Input:**2:     Training set $R=\{\left(y_{1},u_{1}\right),\left(y_{2},u_{2}\right),\ldots ,\left(y_{v},u_{v}\right)\}$3:     Base algorithms $G=\{G_{1},G_{2},\ldots ,G_{s}\}$4:     Meta algorithm *H*5:**Output:** Ensemble Model6:**Start**7:**Step-1:**8:     Train the base algorithms by applying algorithms $G_{i}$ to *R*9:     **For** i=1,2,…,k **do**10:          $E_{i}=G_{i}\left(R\right)$11:     **End For**12:**Step-2:**13:     Generate a new dataset for making predictions *R*14:     **For** j=1,2,…,n **do**15:               Classify the training samples $x_{j}$16:               $z_{ij}=E_{i}\left(x_{j}\right)$17:          **End For**18:          $R=\{y_{j},u_{j}\}$, where $y_{j}=\left\{z_{1j},z_{2j},\ldots ,z_{sj}\right\}$19:     **End For**20:**Step-3:**21:     Train the meta-algorithm *H*22:     H=G(R)23:     **Return** *H*24:**End**

## 4. Experimental Results

The experiments in this study are conducted using Python programming language on Windows 10 @ 1.80 GHZ. The motivation of this study is to propose an ensemble model for the prediction of SKCM disease by utilizing different ML classifiers and to analyze overall survival using Kaplan–Meier and Cox hazard regression models. Initially, we applied three different feature selection methods, i.e., RFE, FFE, and BEF, to select the best features, as discussed in Section 3.

### 4.1. Survival Analysis Clinical Endpoint

The survival analysis with the log-rank test was examined in this study.

#### 4.1.1. Kaplan–Meier Estimator

One of the most popular statistical methods used to estimate the likelihood of an event, such as death, a recurrence of a disease, the emergence of a new disease entity, or an adverse response, is survival analysis [37]. First, we need to understand the survival function to understand survival analysis. For example, consider Y as the random duration taken from the dataset under study as a duration that can be infinite but not a negative value, and the survival function can be denoted as Pro(ti), where Pro(ti) denotes the probability that an event has occurred or not yet at a time ti. It denotes the survival function calculated as Equation (15).

(15) $$\begin{array}{c} \begin{array}{c} \mathrm{Pro}(\mathrm{ti})=x(Y>\mathrm{ti}) \end{array} \end{array}$$

**Figure 5:**
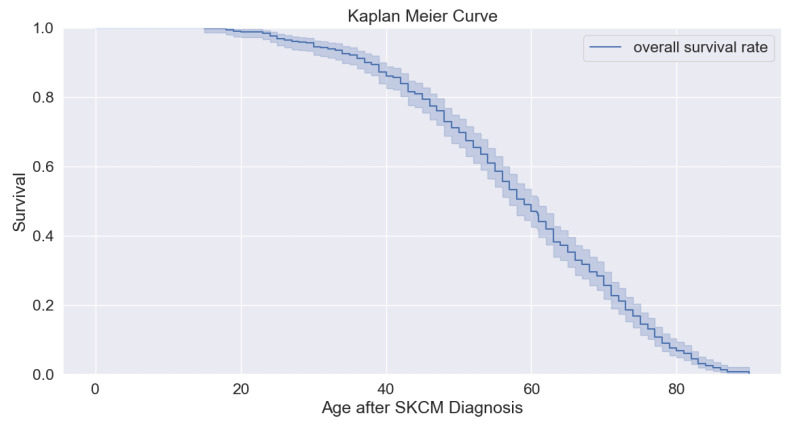
Overall survival (OS) analysis.

The survival analysis can be achieved using the Kaplan–Meier estimator. Re-estimating the survival probability upon each event occurrence can be achieved using the Kaplan–Meier (KM) approach. This non-parametric method does not assume a specific distribution for the outcome variable, such as time. This approach is very simple, and complexity arises as the number of observations increases. We can say that the main idea of the KM approach, depending on the observed event time, is to split the estimation of the survival function into small chunks. The probability for each interval can be formulated using the following Equation (16):

(16) $$\begin{array}{c} \begin{array}{c} \mathrm{Pro}\left(\mathrm{ti}\right)=\prod_{\mathrm{tia}<t}\frac{Z_{\left(a-c_{a}\right)}}{Z_{a}} \end{array} \end{array}$$

where za denotes the number of patients whose lives are at risk at time $t_{i}a$, and $c_{a}$ denotes the number of incidents that occurred in the event at a time $t_{i}a$ (See Figure 5). Figure 5 shows the overall survival analysis of patients. We find the overall survival probability with significance (*p*-value is 0.05) of patients after diagnosing SKCM. The graph shows a higher probability of survival beyond the age of 20 and less than 20 years.

**Figure 6:**
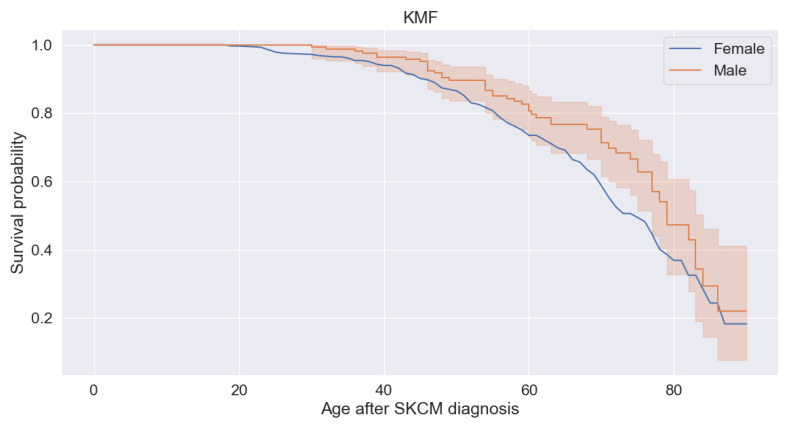
Survival probabilities.

Figure 6 shows the survival probabilities of patients. We find that male patients have a higher probability than female patients. The male patients aged 20 years and below have higher (about 0.8 or 80%) survival probability. Above 80 years and somehow below 80 years, patients have less about (0.2 or 20%) of survival probability.

**Figure 7:**
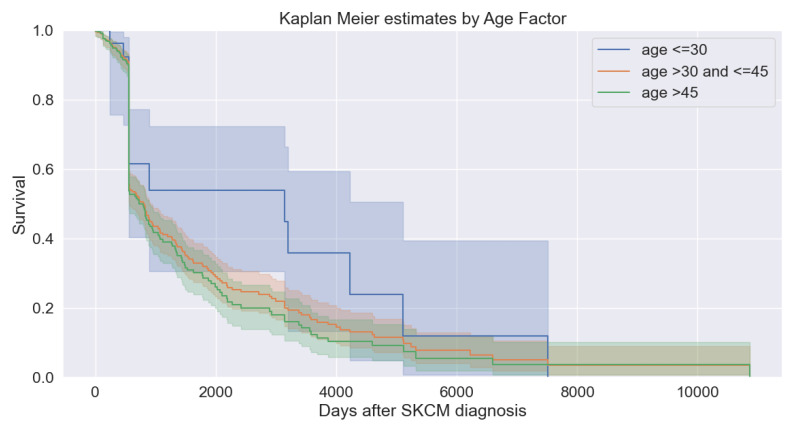
Survival analysis with different age groups.

Figure 7 depicts the survival analysis of different age groups of patients. We analyzed that patients in the age group greater than 30 years and less than or equal to 45 years and those in the age group equal to 45 years are nearly overlapping and have higher (about 0.6 or 60% and above) survival probability. For the patients in the age group less than or equal to 30, the curve shows step-wise increments in the probability starting near the survival probability (0.1 or above) and increasing steadily. All age groups overlap when survival probability reaches between 0.5 or 50% and up to 0.8 or 80%.

**Figure 8:**
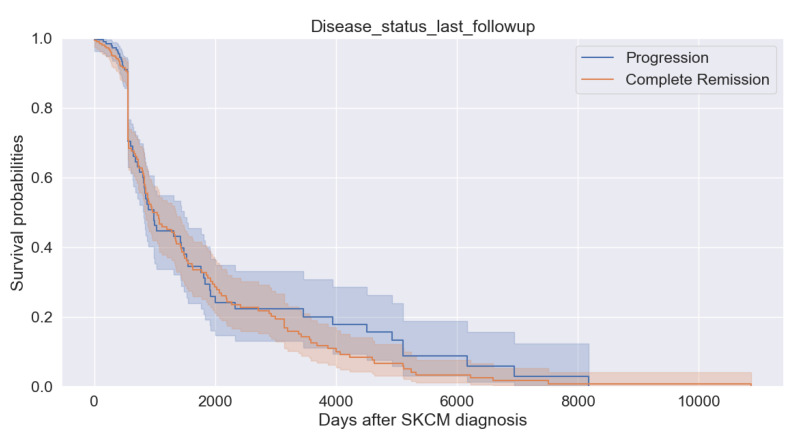
Survival probability with last follow up.

Figure 8 describes the progression and complete remission of survival after diagnosis. For each cohort, there are two survival curves. We can observe that the progression curve increases in a step-wise curve. As the days passed, the probability of progression increased gradually. When survival probability reaches between 70% and 85%, both curves overlap. However, after diagnosis, patients start recovering.

#### 4.1.2. Cox Proportional Hazards Regression Model

The proportional hazards model, developed by David Cox in 1972 [38], uses the proportional risks assumption to produce reliable estimates of covariate effects. The Cox proportional hazards regression model is a semi-parametric approach for estimating weights in a proportional hazard model. It uses gradient descent to fit the data and minimizes errors. The model works by estimating the log hazard of patients as a linear function of their static covariates and a population-level baseline hazard function that changes over time [24]. It can be defined mathematically as Equation (17).

(17) $$\begin{array}{c} \begin{array}{c} E\left(d|e\right)=h_{0}\left(d\right)\exp\left(\sum_{n=1}^{e}y_{n}\left(e_{n}\right)\right) \end{array} \end{array}$$

where

- *d* represents survival time;
- $E\left(d|e\right)$ represents the hazard function determined by a set of factors, i.e., e1,e2,e3,…en;
- $h_{0}\left(d\right)$ defines the baseline hazard function representing event probability when all covariates are zero. Hazard value equals 1 when all $e_{n}$ are zero. The model assumes a parametric form for covariates’ effect on hazard without baseline assumptions;
- $\exp\left(\sum_{n=1}^{b}y_{n}\left(e_{n}\right)\right)$ represents partial hazard as a time-invariant scalar factor that increases or decreases baseline hazard like the intercept in ordinary regression;
- The coefficients (y1,y2,y3,…,yn) measure the impact of covariates on a subject’s hazard. The sign of the coefficient *b* affects the baseline hazard. A positive sign indicates higher risk, whereas a negative sign indicates lower risk. The magnitude of the coefficient b is estimated by maximizing partial likelihood. It assumes a proportional rate ratio throughout the study period, offering increased flexibility. This model can handle right-censored data but not left-censored or interval-censored data directly. The Cox model accepts the following three assumptions:
  1. A constant hazard ratio;
  2. The multiplicativity of explanatory variables;
  3. The independent failure times for individual subjects.

Table 4 describes the Cox proportional hazard regression. We evaluate the Cox hazard model and log-rank test to find the hazard ratio (HR) and significant association among the groups. We find that the value of the hazard ratio HR < 1, which means there is a reduction in the risk. The significance (*p*-value) < 0.05 is considered to find out the association among groups. We observed that the covariate Age at the last followup and interval has *p*-values of 0.02 and 0.03, respectively, less than the significant *p*-value (0.05). We can say that there is an association between the groups.

**Figure 9:**
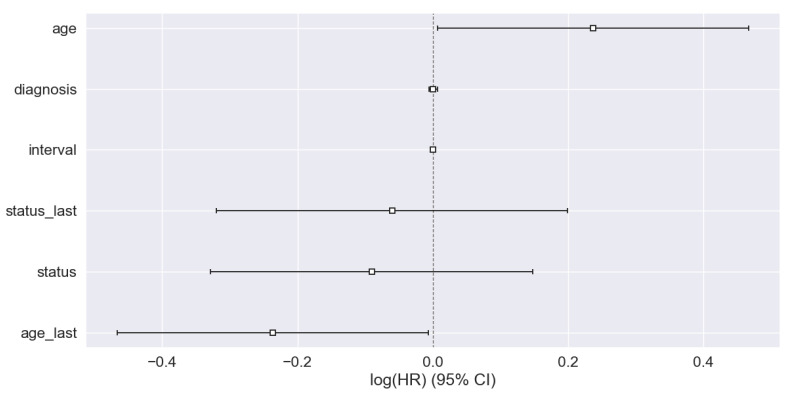
Hazard ratio.

Figure 9 shows the hazard ratio (HR) for different covariates. We find that most of the covariates have HR > 1, meaning there is a risk reduction. Only for one covariate is there no effect, since HR = 1.

#### 4.1.3. ML-Based Ensemble Methods

We trained eight different ML classifiers to create ensemble models. To evaluate the performance of the proposed ML classifiers, we used two performance measures: ROC and confusion matrix. The results are presented in accuracy, precision, recall, F1 score, and ROC curve. The assessment includes four performance metrics: true positive (TP), denoting the accurate classification of ’Positive Reputation’ in positive samples; false positive (FP), representing the misclassification of samples not belonging to the class; true negative (TN), indicating the accurate classification of negative samples; and false negative (FN), signifying the misclassification of samples as positive when they actually belong to the negative class.

Table 5 compares the performance of feature selection methods. We have trained an RF classifier with scoring matric r2 to select the best features. The performance of the RFE method is better than the other two methods.

Table 6 describes the performance of eight different ML algorithms in terms of accuracy, precision, recall, and F1 score on the test dataset. It can be concluded that most of the algorithms achieved the highest accuracy rate of 98%. Only AdaBoost and GNB achieve 97% and 96% accuracy rates. It can be observed that the highest precision achieved by RF is 98%, while the highest recall rate obtained by LR, GB, RF, and light gradient boosting machine (LGBM) is 99%. LR, GB, and LGBM attained the highest F1 score rate. It is noteworthy to mention that the above performance metrics are evaluated on the test dataset.

**Figure 10:**
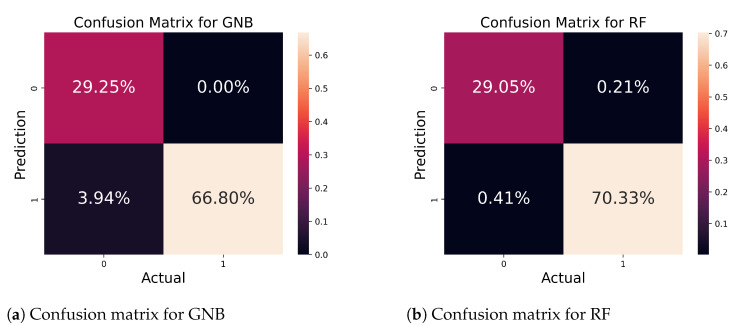
Confusion matrix for GNB and RF algorithms.

In Figure 10a, we present the confusion matrix for the GNB algorithm, showing its robust accuracy in correctly predicting 96.05% out of 482 samples, with only 3.95% samples being predicted inaccurately. On the other hand, in Figure 10b, we demonstrate the confusion matrix for the RF algorithm. There are a total of 482 samples, out of which 99.38% were accurately predicted while 0.62% were incorrectly forecasted. As compared to the other related studies, such as [9,10], our results are highly accurate.

**Figure 11:**
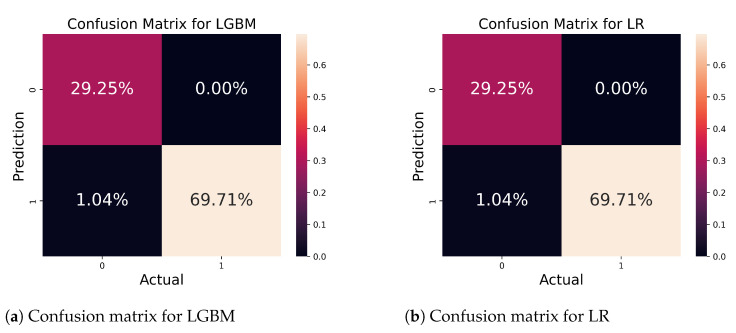
Confusion matrix for LGBM and LR classifiers.

In Figure 11a, we present the confusion matrix for the LGBM; the performance of the classifier was evaluated on a total of 482 samples. The classifier successfully predicted 98.96% samples correctly; only 1.04% samples were incorrectly predicted. Figure 11b depicts the matrix for the LR algorithm. There were 482 samples, out of which 98.96% were precisely predicted while 1.04% of samples were wrongly predicted.

**Figure 12:**
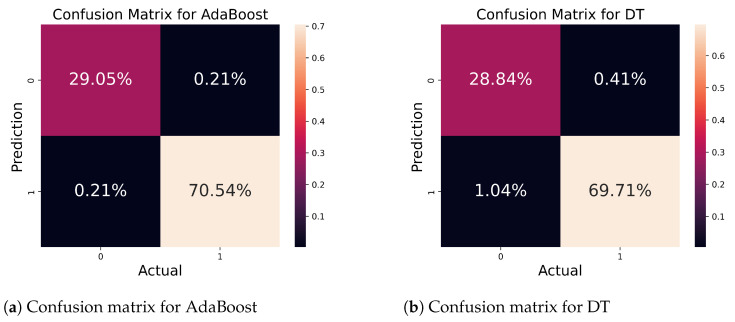
Confusion matrix for AdaBoost and DT classifiers.

In Figure 12a, we show the confusion matrix for the AdaBoost, where the classifiers impressively provide good accuracy by accurately predicting 99.59% out of a total of 100% on 482 samples. In contrast, the classifier wrongly predicted 0.41% samples. Figure 12b illustrates the matrix for the LR algorithm; the algorithm accurately predicted 98.13% out of 100% of samples, and only 1.87% of samples were incorrectly predicted. The classifiers’ performance can be visualized from these insightful representations.

**Figure 13:**
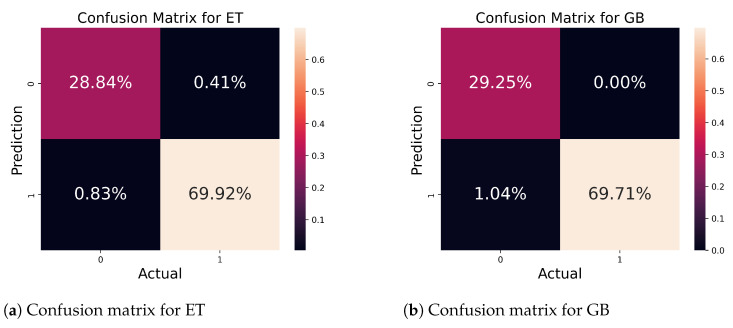
Confusion matrix for ET and GB classifiers.

Figure 13 depicts the confusion matrix for ET and GB algorithms. Figure 13a depicts the matrix for the ET algorithm. The classifier accurately predicted 98.76% out of 100% of samples, and only 1.24% of samples were wrongly predicted. Figure 13b determines the confusion matrix for the GB algorithm. The classifier correctly predicted 98.96% of 100% samples, and the classifier incorrectly predicted 1.04% of total samples.

**Figure 14:**
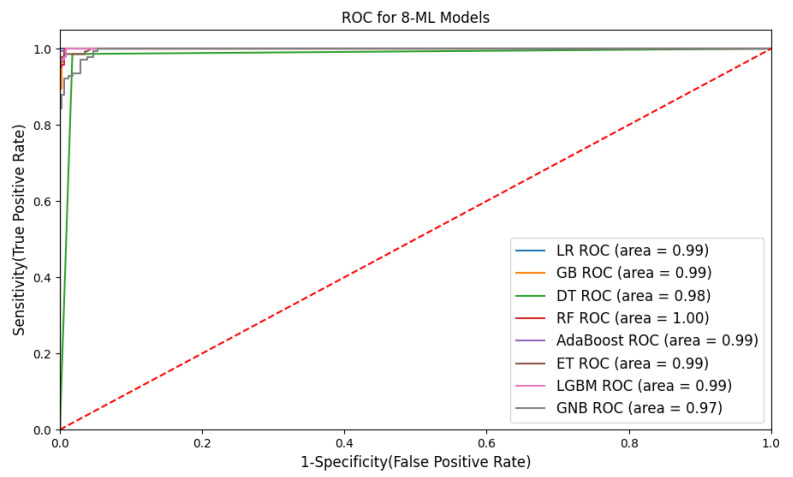
Consolidated ROC curve for ML classifiers.

Figure 14 illustrates the ROC curves for specificity (false positive rate) and sensitivity (true positive rate). The model can be classified or perform well if the ROC curve is turned to the upper left corner. Most of the classifiers turn toward the left upper corner, which means the classifiers perform well. From the below graph, we can say that LR, RF, AdaBoost, and LGBM achieve the highest accuracy, which is 0.99, whereas GB, DT, and extra tree achieve an accuracy of 0.98. Only the NB classifiers attained 0.97 accuracy. It can be concluded that there is a slightly small difference in the accuracy of different classifiers.

These ensemble methods were generated by training the above eight base ML classifiers. It can be observed that the stacking and voting method achieved the highest accuracy rate, which is 99%, as illustrated in Table 7. The highest precision rate recorded is 98% and is obtained by the stacking method. The voting method attained the highest recall rate, as well as F1 score, which was 99%.

**Figure 15:**
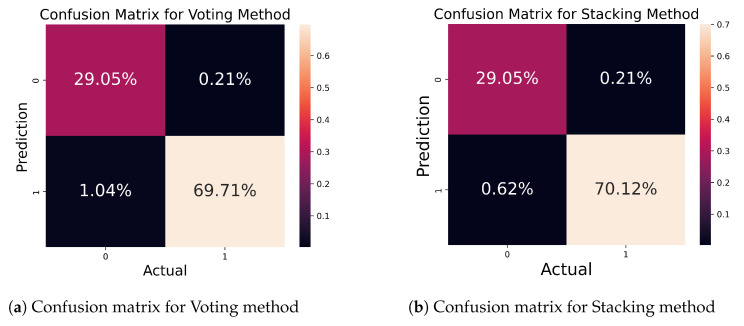
Confusion matrix for ensemble method.

Figure 15 portrays the matrix for the voting and stacking ensemble methods. From Figure 15a, out of 100% samples, 98.76% of samples were accurately predicted by this method, while 1.24% of samples were wrongly predicted. Figure 15b shows the matrix for the stacking method. This method correctly predicted 99.17% of samples, and only 0.83% of samples were predicted incorrectly.

**Figure 16:**
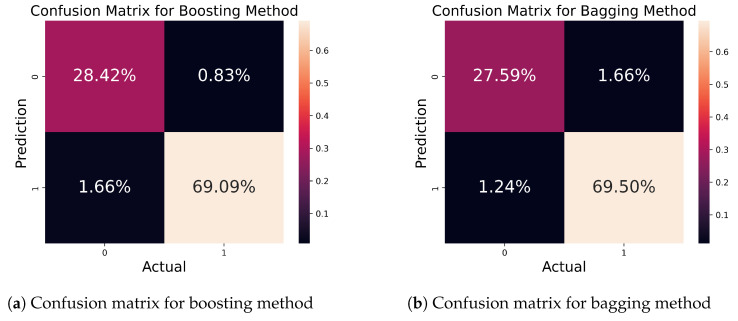
Confusion matrix for ensemble model.

Figure 16 portrays the matrix for the boosting and bagging ensemble methods. From Figure 16a, out of a total of 100% samples, 97.51% were correctly predicted while 2.49% were wrongly predicted. Figure 16b shows the matrix for the bagging method. Here, 97.09% of samples were predicted correctly, and only 2.91% of samples were wrongly predicted.

### 4.2. Comparative Study

**Figure 17:**
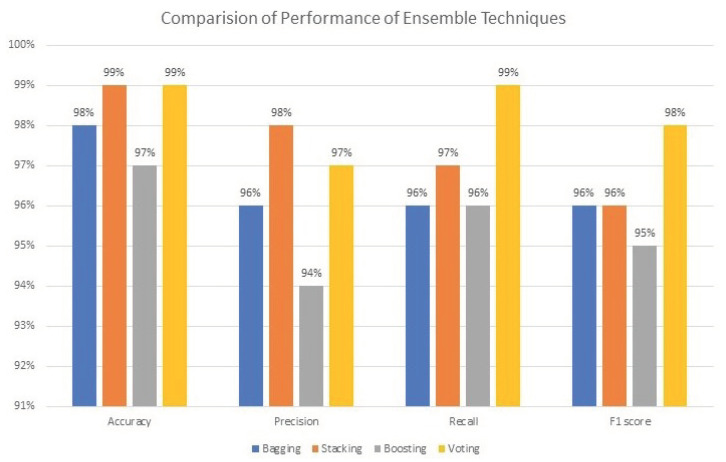
Performance of ensemble methods.

**Figure 18:**
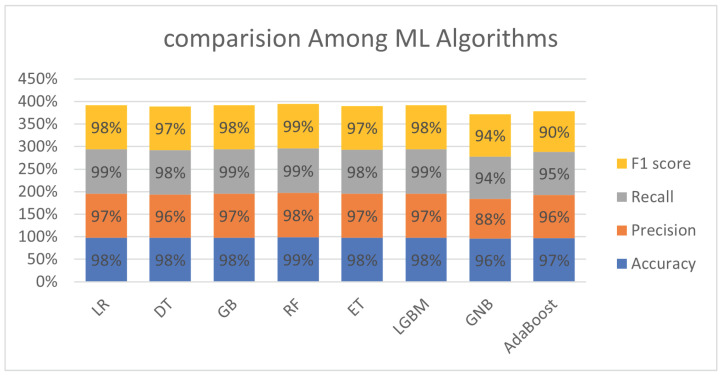
Comparison of different ML classifiers.

This study aims to propose an ensemble model for the prediction of SKCM cancer by utilizing ML classifiers. To create an ensemble model, we trained four different ensemble methods with eight different ML classifiers. The performance of ML classifiers and ensemble methods is compared and discussed below (see Figure 17 and Figure 18).

Table 8 compares the performance of baseline classifiers and ensemble methods. We find that the stacking and voting ensemble methods achieved the highest performance as compared to baseline classifiers. Figure 17 illustrates the performance of different ensemble methods. The methods perform well. Furthermore, Figure 17 shows that stacking and voting outperform as compared to other methods.

Figure 18 represents the comparison among ML classifiers. The performance was evaluated in accuracy, precision, recall, and F1 score. However, all classifiers perform well, but the RF classifier outperforms all others. The main purpose of this study is to propose an ML-based ensemble method for the prediction of SKCM and to analyze the survival probability using the Kaplan–Meier and Cox proportional hazards regression models.

### 4.3. Baseline Classifiers Standard Error

Table 9 illustrates the standard error computed by eight baseline classifiers. This measure gauges the reliability of performance metrics like accuracy, precision, recall, and F1-score. Notably, an inverse relationship between accuracy and standard error was observed. Analysis of the table indicates that classifiers exhibiting the lowest standard error demonstrate greater stability and consistency in their performance across various metrics.

**Figure 19:**
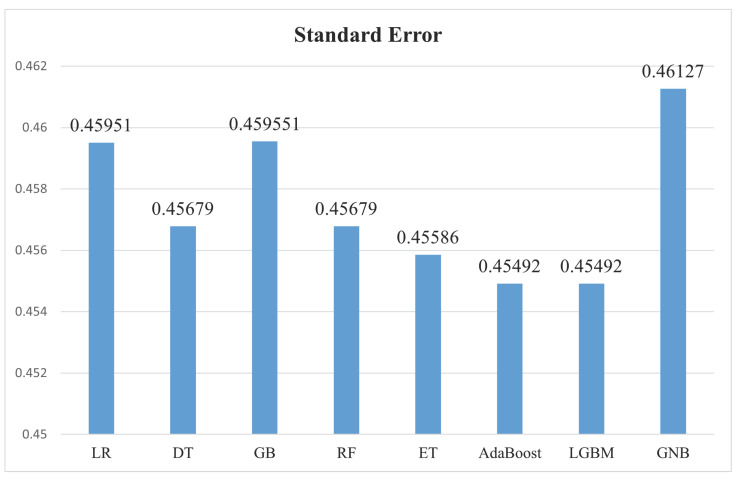
Baseline classifiers standard error.

Figure 19 displays the standard error associated with each baseline classifier. This metric offers crucial insights into the stability of model performance. Among the classifiers, GNB predicts a slightly higher standard error than others. Nonetheless, there are nuanced differences among the standard errors across the algorithms. AdaBoost and LGBM classifiers notably showcase the least standard error. A smaller standard error signifies a higher likelihood of consistent performance across various cross-validations, whereas higher standard errors suggest more variability in performance.

Table 10 showcases the standard error forecasted by individual ensemble methods. Among these methods, the lowest standard error, at 0.45397, is observed in the bagging method, indicating superior performance compared to the other ensemble techniques.

**Figure 20:**
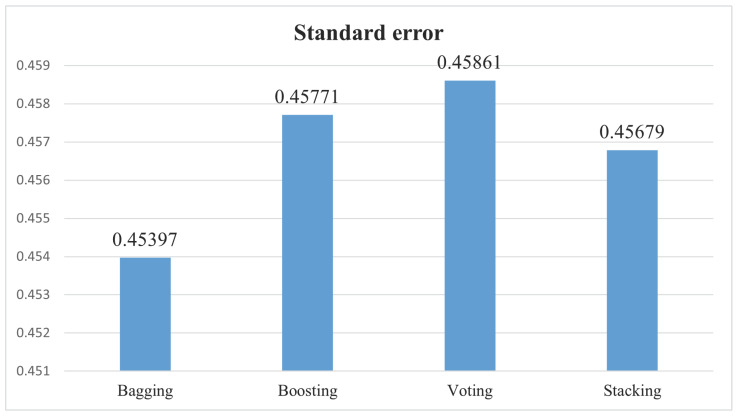
Ensemble methods standard error.

Figure 20 illustrates the standard error produced by the four ensemble methods. A higher standard error signifies increased variability in the model’s performance. Among these methods, the voting method attains the highest standard error of 0.45861, indicating lower consistency in model performance. Conversely, the bagging method achieves the lowest standard error at 0.45397, signaling superior model performance.

### 4.4. Discussion

Skin cancer is considered one of the most dangerous types of cancer. Many studies focus on early detection, treatment approaches, and suggesting prevention techniques. Numerous studies in the past have delved into these aspects, with a notable focus on employing ML and DL techniques that have yielded promising results for early detection and prognosis of the disease. Previous studies mainly focused on survival analysis and early detection using DL techniques. Our study proposes four ensemble methods (stacking, bagging, boosting, and voting) to predict SKCM and to analyze survival probability using KM and Cox proportional hazards regression models. In constructing our ensemble methods, which encompass stacking, bagging, boosting, and voting, we meticulously trained and tested eight baseline classifiers: RF, LR, DT, GB, ET, Adaboost, LightGBM (LGBM), and GNB. The performance of these methods was rigorously evaluated using a suite of metrics, including accuracy, precision, recall, F1 score, confusion matrix, and ROC curve. Remarkably, our results demonstrate a pinnacle of accuracy, reaching an impressive 99%, achieved by the stacking and voting ensemble methods. This exhibits the robustness and efficacy of the ensemble techniques employed in our study. Notably, among the individual algorithms, RF emerged as the top-performing classifier, depicting superior predictive capabilities. This exceptional performance across multiple metrics shows the potential applicability of our proposed ensemble methods in the realm of SKCM prediction. The high accuracy rates, especially with stacking and voting methods, suggest a synergistic enhancement of predictive power by combining diverse classifiers. Such findings hold significant implications for the development of more reliable and accurate predictive models in the context of skin cancer.

## 5. Conclusions

In this paper, Kaplan–Meier and Cox proportional hazards regression models are used to analyze overall survival, and ML-based ensemble methods are proposed to predict SKCM. Five distinct datasets using transcriptomic technologies were collected. To choose the best features, three distinct feature selection methods, i.e., REF, FFE, and BFE, were used. We trained and compared four ensemble approaches (stacking, bagging, boosting, and voting) using eight baseline classifiers (RF, DT, GNB, AdaBoost, GB, LR, ET, and LGBM). The performance of ensemble methods was evaluated with the help of the ROC curve, confusion matrix, accuracy, precision, recall, and F1 score. The overall performance of RF was good as compared to other classifiers. The recorded performance of the algorithms shows a slight variation. Voting and stacking strategies scored the best among ensemble techniques. The highest ROC was achieved using RF, LR, AdaBoost, and LGBM, which was 0.99. The RF classifier achieved the best accuracy, which was 99%, and the stacking and voting method achieved the highest accuracy rate, which was 99%. Finally, this study is limited to a specific dataset, which can be evaluated on various datasets to achieve better results.

### Future Work

We will investigate deep learning methods for skin cancer early detection and prognosis in the future. We will investigate other multi-omic technologies in this area and investigate various skin cancers to further find out ways for early detection of diseases. 

## Figures and Tables

**Table 1:** Summarized related work and its limitations.

Ref.	Method	Study Area	Dataset	Results	Limitations
[25]	A CNN-based melanoma skin cancer detection	Skin cancer	Open access dataset	95%	No multi-omics exploited
[26]	DL-based skin cancer detection system	Skin cancer	HAM 10000	91%	The accuracy of proposed study is poor
[27]	DL-based melanoma detection	Skin cancer	HAM 10000	86%	Various models and datasets call for different hyperparameter settings
[28]	CNN-based skin cancer detection	Skin cancer	HAM 10000	91.93%	The limited size of the datasets employed in this study may have led to local optimizations
[29]	A DL-based framework for the multi-classification of skin cancer using dermoscopy images	Skin cancer	ISIC 2019	92%	Lower accuracy
[9]	Immune cell infiltration pattern of CM	SKCM	-	-	The study does not utilize any ML/DL algorithms to show a better performance
[10]	DL-based short-term survival of cutaneous malignant melanoma (CMM)	SKCM	RNA-seq	91%	Lower accuracy
[30]	The mRNA expressions of m5C regulators in colorectal cancer tissues	Various cancer types	RNA-seq	-	Performance metrics were not evaluated
[31]	DL-based multiclass skin cancer diagnosis	Skin cancer	HAM 10000 ISIC	96.26%	The accuracy of this study is lower

**Table 2:** Notations and descriptions.

Notation	Description
*A*	Input set of features for backward selection
$B_{0}$	Initialize the function with a full set of features
*w*	Subset of output features
*r*	A finite set of input features
$D_{w}$	Output set of features
$T^{+}$	Selection criteria function
$g_{i}$	Subset of output features
*L*	The desired set of features
*m*	A finite set of output features
*X*	Input set features for forward selection
$X_{0}$	Initialize the function with an empty set
$p^{+}$	Selection criteria function
*h*	The desired set of features
$Z_{t}$	Output set of features
$z_{b}$	Subset of output features
*t*	Size of a subset of output features
Pro	The likelihood of the event occurred
ti	Time at which event occurred or did not occur
*Y*	Random duration of survival function
za	Number of patients
ca	Number of incidents
tia	Life risk at a time
*d*	Survival time
E(d∣e)	Hazard function
$e_{n}$	Set of factors
$h_{0}\left(d\right)$	Baseline hazard function
$y_{n}$	Measure of the impact of covariates on a subject’s hazard

**Table 3:** Dataset description.

Class	Records per Class	Features
Donor	471	9
Simple_somatic_mutation	1,048,576	12
Copy-number_soamtic_mutation	377,735	3
Mirna _seq	369,409	5
Specimen	947	2
Total Records	1,797,138	31

**Table 4:** Cox hazard proportional method.

Covariate	Coef	Exp (Coef)	Se (Coef)	Coef Lower (95%)	Coef Upper (95%)	Exp Coef Lower (95%)	Exp Coef Upper (95%)	*z*	*p*	log2 (p)
Sex	0.02	1.02	0.10	−0.17	0.21	0.84	1.23	1.02	0.10	−0.17
Status	−0.12	0.98	0.19	−0.39	0.35	0.68	1.41	0.98	0.19	−0.39
Disease status last followup	−0.00	0.98	0.10	−0.21	0.17	0.81	1.18	0.98	0.10	−0.21
Age at last followup	−0.50	0.61	0.02	−0.55	−0.46	0.58	0.63	0.61	0.02	−0.55
Diagnosis	−0.00	1.00	0.00	−0.01	0.00	0.99	1.00	1.00	0.00	−0.01
Interval	0.49	1.64	0.03	0.44	0.54	1.56	1.72	1.64	0.03	0.44

**Table 5:** Feature selection methods and scores.

Feature Selection Methods	Score
Backward Feature Elimination	0.99993
Forward Feature Elimination	0.99988
Recursive Feature Elimination	0.99400

**Table 6:** Performance metrics of ML algorithms.

ML Algorithms	Accuracy	Precision	Recall	F1 Score
LR	98%	97%	99%	98%
DT	98%	96%	98%	97%
GB	98%	97%	99%	98%
RF	99%	98%	99%	99%
ET	98%	97%	98%	97%
AdaBoost	97%	96%	95%	90%
LGBM	98%	97%	99%	98%
GNB	96%	88%	94%	94%

**Table 7:** Performance metrics of ensemble methods.

Ensemble Methods	Accuracy	Precision	Recall	F1 Score
Bagging	98%	96%	96%	96%
Stacking	99%	98%	97%	96%
Boosting	97%	94%	96%	95%
Voting	99%	97%	99%	98%

**Table 8:** Performance metrics of ML classifiers.

ML Classifiers	Accuracy	Precision	Recall	F1 Score
RF	99%	98%	99%	99%
GNB	96%	88%	94%	94%
LR	98%	97%	99%	98%
DT	98%	96%	99%	98%
GB	98%	97%	99%	98%
Adaboost	97%	96%	95%	90%
Extratree	98%	97%	98%	97%
LGBM	98%	97%	99%	98%
Bagging	98%	96%	96%	96%
Stacking	99%	98%	97%	96%
Boosting	97%	94%	96%	95%
Voting	99%	97%	99%	98%

**Table 9:** Baseline classifiers standard error.

Baseline Classifiers	Standard Error
LR	0.45951
DT	0.45679
GB	0.459551
RF	0.45679
ET	0.45586
AdaBoost	0.45492
LGBM	0.45492
GNB	0.46127

**Table 10:** Ensemble methods standard error.

Ensemble Methods	Standard Error
Bagging	0.45397
Boosting	0.45771
Voting	0.45861
Stacking	0.45679

## Data Availability

The data in this study can be provided upon request.
